# Asymptomatic Esophageal Necrosis in a Patient with Recent COVID-19: The First Case Diagnosed through Autopsy

**DOI:** 10.3390/medicina59010154

**Published:** 2023-01-12

**Authors:** Ionuț Isaia Jeican, Patricia Inișca, Bogdan Alexandru Gheban, Vlad Anton, Costel Vasile Siserman, Codrin Rebeleanu, Maria Aluaș, Carmen Bianca Crivii, Silviu Albu, Veronica Trombitaș

**Affiliations:** 1 Department of Anatomy and Embryology, Iuliu Hatieganu University of Medicine and Pharmacy, 400006 Cluj-Napoca, Romania; 2 Department of Pathology, County Emergency Hospital, 330084 Deva, Romania; 3 Department of Histology, Iuliu Hatieganu University of Medicine and Pharmacy, 400349 Cluj-Napoca, Romania; 4 Department of Medical Biochemistry, Iuliu Hatieganu University of Medicine and Pharmacy, 400349 Cluj-Napoca, Romania; 5 Institute of Legal Medicine, 400006 Cluj-Napoca, Romania; 6 Department of Legal Medicine, Iuliu Hatieganu University of Medicine and Pharmacy, 400006 Cluj-Napoca, Romania; 7 Department of Oral Health, Iuliu Hatieganu University of Medicine and Pharmacy, 400012 Cluj-Napoca, Romania; 8 Department of Head and Neck Surgery and Otorhinolaryngology, University Clinical Hospital of Railway Company, Iuliu Hatieganu University of Medicine and Pharmacy, 400015 Cluj-Napoca, Romania

**Keywords:** COVID-19, esophageal necrosis, autopsy

## Abstract

Acute esophageal necrosis is a rare condition, characterized by a distinctive endoscopic/necropsic image–circumferential black area of the esophagus. This paper presents a case of a 78-year-old patient with recent history of a severe form of COVID-19 (2 months previously), with multiple comorbidities, which presents sudden death in hospital. Anatomic-pathological autopsy showed extensive esophageal necrosis, pulmonary thromboses, and coronarian and aortic atherosclerosis. The histopathological examination revealed necrosis of the esophageal mucosa and phlegmonous inflammation extended to the mediastinum, chronic pneumonia with pulmonary fibrosis, viral myocarditis, papillary muscle necrosis, and pericoronary neuritis. Thromboses and necroses were identified also in the liver, pancreas, and adrenal glands. Post-COVID-19 thromboses can manifest late, affecting various vascular territories, including esophageal ones. Their clinical picture may be diminished or absent in elderly and/or diabetic patients.

## 1. Introduction

Acute esophageal necrosis (AEN) is a rare condition, characterized by a distinctive endoscopic/necropsic image–circumferential black area of the esophagus, tissue hypoxia associated with debilitated physical states being the underlying etiology [1]. By disrupting the inflammatory and coagulation mechanisms, including vasculopathy, COVID-19 causes tissue hypoxia, which can lead to AEN [2]. In addition, the risk of thrombosis after COVID-19 is already known [3]. Through a combination of thrombocytopenia, prolonged prothrombin time and elevated D-dimer, COVID-19 is thought to cause venous and arterial thrombotic complications similar to disseminated intravascular coagulation (DIC) or thrombotic microangiopathy [4].

The aim of the study was to analyze the autopsy results of a 78-year-old male patient, with a recent history of a severe form of COVID-19 (2 months previously), with clinically unmanifested extensive esophageal necrosis. To our knowledge, this is the first case with esophageal necrosis in a COVID-19 context diagnosed and analyzed through autopsy.

## 2. Case Presentation

This study was carried out with the consent of the patient’s family and approved by the Ethics Committee of Iuliu Hatieganu University of Medicine and Pharmacy, Cluj-Napoca, Romania, No. 63/01 March 2022.

A 78-year-old patient with recent history of a severe form of COVID-19 (2 months previously), post-COVID-19 pulmonary fibrosis, and Clostridium difficile enterocolitis, requests ambulance service for rest dyspnea, cough, marked asthenia, and diarrhea. The patient has multiple comorbidities: type 2 insulin-requiring diabetes, stage 4 chronic renal failure, and major right bundle branch block. The patient was receiving treatment at home with apixabanum 2 × 2.5 mg/day, ipratropium bromide, insulin glargine 30 IU/day, ivabradine 2 × 5 mg/day, and probiotics.

During the objective examination, the patient is hypersthenic, drowsy, with central type cyanosis, accentuated bilateral vesicular breath sounds, blood pressure 90/50 mmHg, ventricular rate 105 bpm, peripheral oxygen saturation 87% in atmospheric air, and afebrile at the time of examination.

Biological investigations reveal leukocytosis (32.32 × 109/L) with neutrophilia (27.15 × 109/L, 84%), coagulopathy-prolonged Quick time (15.8 s), low prothrombin index (68.6), prolonged international normalized ratio INR (1.95), nitrogen retention (creatinine 2.17 mg/dL, eGFR 28.12 mL/min, urea 111.45 mg/dL), hyperglycemia (193 mg/dL), C-reactive protein 96.67 mg/L, procalcitonin 4.17 ng/mL, LDH 302 U/L, mild hyponatremia (134.2 mmol/L), and Clostridium difficile toxins A and B were negative in the stool sample.

Combined throat/nasal sampling (with collection device produced by Sanimed International Implex, Bucharest, Romania) was performed for real-time PCR (RT-PCR) SARS-CoV-2 (QuantStudioTM5 analysis method with TaqPathTM COVID-19 CE-IVD RT-PCR Kit, Thermo Fisher Scientific, Pleasanton, CA, USA). The result was negative. Respiratory secretions were not collected for microbiological diagnosis.

The established diagnosis is aggravated chronic respiratory failure, acute enterocolitis, type 2 insulin-requiring diabetes, and chronic renal failure stage 4. The patient is hospitalized and treatment with antibiotic (Metronidazole 3 × 250 mg/day), antidiarrheals (Furazolidone), mask oxygen therapy with a flow rate of 4 L/min, and hydro-electrolytic rebalancing is started in addition to the treatment followed at home and previously mentioned.

After 24 h, the patient shows favorable clinical evolution and feeds himself, but the biological parameters remain around the same values (peripheral oxygen saturation 94% with 4 L oxygen by mask, blood pressure 110/65 mmHg).

**Figure 1:**
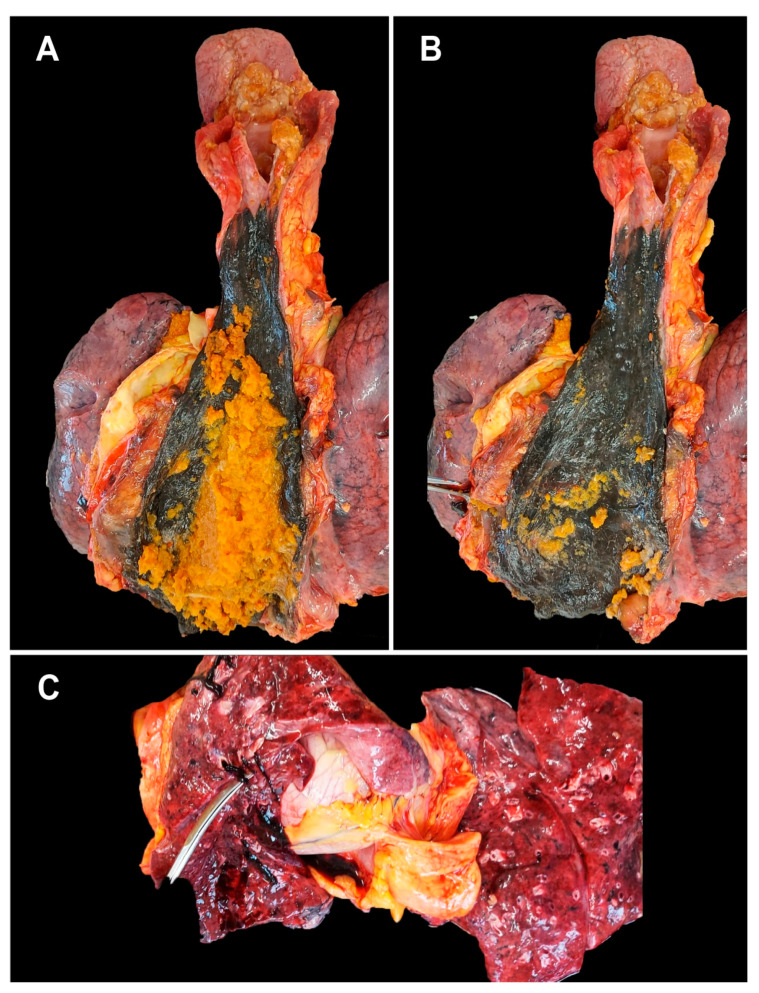
Macroscopic aspects at autopsy: (**A**)—esophageal food stasis; (**B**)—dilated and necrotic esophagus in the lower 2/3; (**C**)—well-formed pulmonary thrombus, rolling on the tip of the clamp.

48 h after admission, the patient presents sudden death—mechanically and pharmacologically irresuscitable cardio-respiratory arrest. According to the applicable Romanian legislation (Law 104/2003), to establish the cause of death, an anatomic-pathological autopsy is performed on the patient. Macroscopically, the following pathological aspects are observed: dilated esophagus, with an increased amount of food content (Figure 1A), the esophageal mucosa shows an extensive, but well-defined, circumferential black area (Figure 1B). The stomach contains food bolus mixed with partially digested blood.

The lungs have a purple-reddish color, and the surface of the section shows a foamy pink serous liquid and dark blood. Pulmonary thromboses are identified (Figure 1C).

The myocardium shows elastic consistency, with numerous small whitish striae, diffusely arranged, and an inhomogeneous area bordered by a hyperemic thickening at the level of the lateral wall of the left ventricle. The lumen of the left coronary artery is narrowed by atherosclerotic plaques. The aortic endothelium presents numerous atherosclerotic plaques in different stages of development.

**Figure 2:**
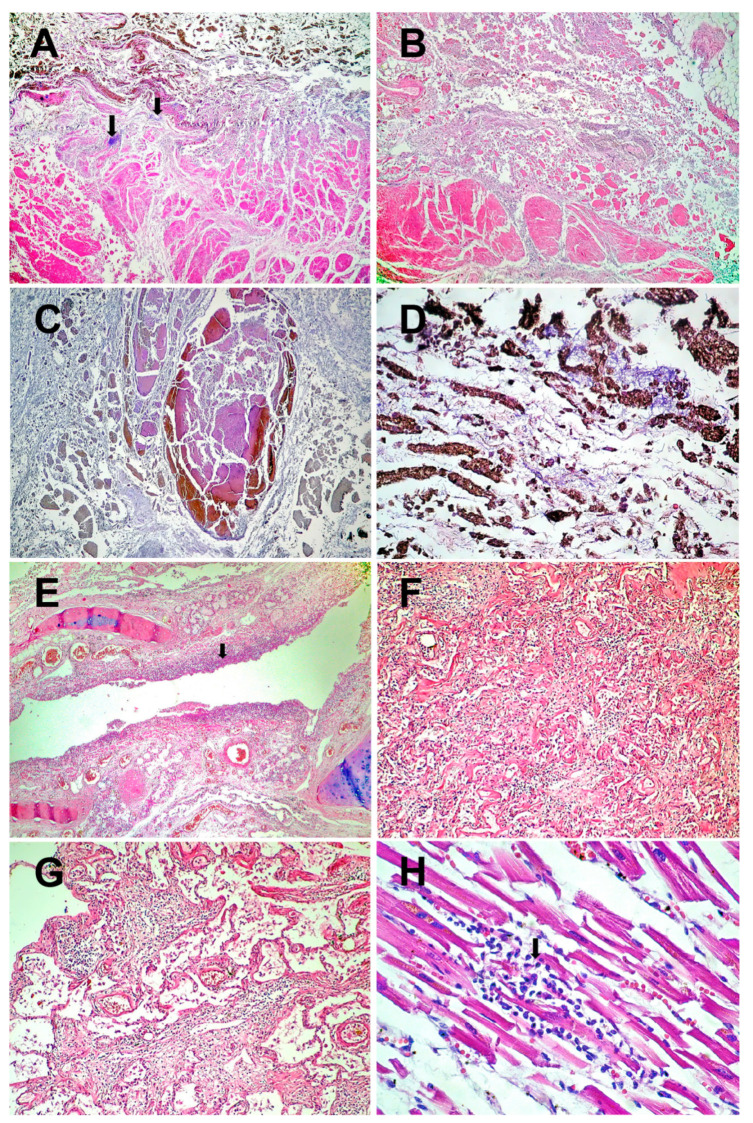

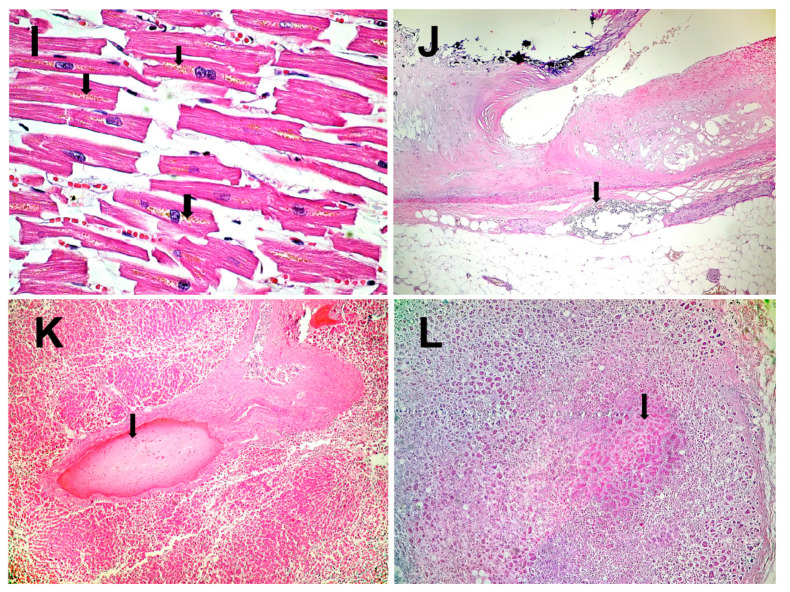
Histopathological results: (**A**)–Esophageal wall with necrotic mucosa invaded by fungal colonies (arrows) and acute transparietal inflammation (HE × 40). (**B**)–Acute phlegmonous inflammation of the esophageal wall, extending to the mediastinum (HE × 40). (**C**)–Thrombosis of the veins in the necrotic esophagus (HE × 40). (**D**)–Necrosis of the mucosa of the lower esophagus colonized by fungi (HE × 200). (**E**)–Necrosis of the bronchial epithelium (arrow) and inflammation of the mucosa (acute bronchitis) (HE × 40). (**F**)–Chronic pneumonia (HE × 100). (**G**)–Residual alveolar and interstitial inflammation (HE × 100). (**H**)–Viral myocarditis (HE × 200), lymphocytes (arrow). (**I**)–Fragments of hypertrophic myocardial fibers with lipofuscin in the cytoplasm (arrows) (HE × 400). (**J**)–Pericoronary neuritis (arrow). Atheromatous coronary artery with calcifications (HE × 40). (**K**)–Liver with intrahepatic thrombosis of the large portal veins (arrow). In the background, nutmeg liver of chronic stasis (HE × 40). (**L**)–Microinfarct in adrenal gland (arrow) (HE × 40).

The histopathological examination revealed necrosis of the esophageal mucosa (Figure 2A) and phlegmonous inflammation extended to the mediastinum (Figure 2B). Extensive venous thromboses are present in the esophageal wall (Figure 2C). Fungal colonies are located on the surface of the esophageal mucosa (Figure 2D).

At the level of the respiratory system, necrotizing bronchitis (Figure 2E), BALT hyperplasia, pulmonary thromboses, chronic pneumonia with pulmonary fibrosis (Figure 2F–G), and acute focal pulmonary edema are found.

At the cardiac level, viral myocarditis (Figure 2H), fragmentation of myocardial fibers (Figure 2I), papillary muscle necrosis, and pericoronary neuritis (Figure 2J) are observed.

Thromboses and necroses are identified in the liver, pancreas, and adrenal glands (Figure 2K–L). This section may be divided by subheadings. It should provide a concise and precise description of the experimental results, their interpretation, as well as the experimental conclusions that can be drawn.

## 3. Discussion

AEN is four times more commonly found in men compared to women, is more common in older patients, and is often associated with multiple comorbidities [5]. The most common comorbidities are diabetes mellitus (38% of cases), hypertension (37% of cases), and chronic kidney disease (16% of cases) [6]. Our patient suffered from both diabetes mellitus and chronic kidney disease.

AEN is thought to be caused by a combination of factors, including ischemic injury, weakened mucosal barrier systems, and backflow injury (caused by gastric acid, pepsin, and other gastric contents) resulting from gastric reflux [7]. Diabetic neuropathy of the autonomic nervous system can often lead to gastroparesis and intensification of gastro-esophageal reflux disease, which may, at least in some cases, explain the link between diabetes and AEN [8]. We also believe that diabetic neuropathy explains the complete absence of esophageal syndrome in our patient’s clinical picture. COVID-19-related thrombosis may contribute to the ischemic injury of the distal esophagus, constituting a further risk factor for AEN [4].

The possible mechanisms by which COVID-19 can cause coagulopathies, including potentially fatal thrombosis, were previously reviewed [4]. A systematic review and meta-analysis of prophylactic anticoagulants administered to ambulatory and discharged COVID-19 patients concluded that prophylactic anticoagulant treatment of all patients’ weeks after disease onset is probably not beneficial [9]. However, one randomized controlled trial showed a 3-fold reduction in the risk of developing some form of symptomatic or fatal thromboembolism in the 5 weeks following discharge from the hospital for patients receiving extended thromboprophylaxis, implying that COVID-19 patients are at risk of thrombosis many weeks from disease onset and well after concluding hospital treatment [10].

COVID-19 can lead to neurological complications, which are thought to be caused either directly, by SARS-CoV-2 infecting neurons, or indirectly, via the inflammatory and prothrombotic effects of the disease [11]. Although rare, the most reported types of neuritis associated with COVID-19 are optic neuritis [12,13] and vestibular neuritis [14]. We have not identified previous reports of pericoronary neuritis in the context of COVID-19 (Figure 2J). COVID-19 can also be associated with cardiovascular autonomic neuropathy [15], although the pathophysiological mechanisms of cardiovascular autonomic dysfunction following COVID-19 are not fully understood [16].

The histopathological identification of viral myocarditis (Figure 2H) two months after COVID-19 is another interesting aspect of our study. In the absence of a molecular confirmation, in the etiology of this myocarditis we can consider both SARS-CoV-2 and other viruses. COVID-19-related myocarditis may result from the viral infection of ACE2-expressing cardiomyocytes or from T-cell-mediated cytotoxicity [17]. Studies have shown lymphocyte and macrophage infiltration of myocardial tissue, with some confirming the presence of viral particles in cardiac macrophages, but most have not identified viral particles inside cardiomyocytes. The study of myocarditis in COVID-19 is further complicated by the difficult diagnosis of the condition, as the gold standard diagnostic tool (excepting autopsy in deceased patients) remains endomyocardial biopsy, a procedure not routinely performed [18].

Myocarditis was not only identified as the cause of death in multiple COVID-19 patients [19] but also as a concerning type of COVID-19-induced long-term cardiovascular complication [20]. Although myocarditis remains a relatively rare condition, one study found a 16-fold increased risk of myocarditis in COVID-19 patients, with approximately 10% of myocarditis cases occurring one to two months after the COVID-19 diagnosis [21]. Another study also highlighted the increased risk of myocarditis two to three months following COVID-19, but its methodology has been called into question as it may have overestimated myocarditis cases [22].

The digestive tract is colonized by opportunistic fungi. Few cases of transmural necrosis candidiasis have been reported and are associated with serious immunosuppression and neutropenia or other comorbid conditions, such as patients on hemodialysis [23]. In our patient, given the presence of thromboses in multiple organs (esophagus, lung, liver, pancreas, and adrenal glands), it is unlikely that the fungal infection was the etiological factor triggering AEN. We can think of the secondary fungal colonization of the necrotic esophagus as both an antemortem event (during the time between the onset of ischemia and the extension of the lesions to the mediastinum) and postmortem (within the 12 h until the time of autopsy).

Considering the sudden aspect of our patient’s death, it was caused directly by the heart; papillary muscle necrosis identified histopathologically argues for myocardial infarction, chronologically installed secondary to AEN. COVID-19 condition can be seen as a determining factor of AEN through changes in the microenvironment that lead to disease.

Previously reported cases of AEN associated with COVID-19 are summarized in Table 1. Among the four reported cases (57–76-year-old), only in one case [2] AEN manifested post-COVID-19, one month later. All cases are male patients, and all presented clinical pictures of digestive hemorrhage, especially melena (3 of 4 patients). All previously reported patients were diagnosed with AEN through digestive endoscopy. Despite the treatment, of the four patients, two died.

## 4. Conclusions

The contrast between the extension of esophageal lesions to the mediastinum and the total absence of esophageal clinical manifestations can be explained by diabetic neuropathy of the autonomic nervous system.

This study draws attention to the reality that post-COVID-19 thromboses can manifest late, despite anticoagulant treatment, affecting various vascular territories, including esophageal ones. The clinicians must pay attention to the clinical picture of post-COVID-19 thromboses which may be diminished or absent in elderly and/or diabetic patients.

## Figures and Tables

**Table 1:** Esophageal necrosis reported in COVID-19 patients.

Authors	Patient/ COVID-19 Status	Clinical Picture	Endoscopy	Histology	Treatment and Clinical Evolution
Deliwala et al. [24]	58-year-old man, COVID-19 active.	Melena, severe anemia, and renal failure.	Distal esophageal necrosis, hiatal hernia, and gastroduodenal ulcerations.	N/A	Packed red blood cell transfusion; esomeprazole, octreotide, and ceftriaxone followed by omeprazole and sucralfate after discharge. Patient recovered.
Mustafa et al. [2]	A 57-year-old man, one month post COVID-19.	Melena, diabetes mellitus, acute kidney injury, and leukocytosis with neutrophilia.	Two wide-based distal esophageal ulcers without active bleeding.	Ulcerated squamous mucosa with extensive necrosis extending to the muscularis propria and coccoid bacterial colonies with rare fungal forms suggestive of Candida.	Fluconazole and pantoprazole, favorable evolution.
Rahim et al. [25]	74-year-old man, COVID-19 active.	Severe complicated pneumonia, melena, and anemia. History of hypertension, type II diabetes mellitus, and hyperlipidemia.	Extensive necrosis of the lower esophagus suggestive of AEN.	N/A	Massive transfusion protocol and pantoprazole, followed by IV fluids and maintenance pantoprazole along with vancomycin, meropenem, and micafungin for sepsis. Patient died 3 days later due to cardiac arrest caused by CODID-19-related respiratory failure.
Abu-Abaa et al. [26]	76-year-old man, COVID-19 active.	Hyperactive delirium, acute kidney injury, hypotension, hematemesis, and acute pancreatitis. History of hypertension, stage 4 severe chronic kidney disease, and gastroesophageal reflux disease.	Severe diffuse ulcerative esophagitis, circumferential submucosal hemorrhages, and gangrenous changes suggestive of ischemic injury.	N/A	Proton pump inhibitor (unspecified) treatment and peripheral parenteral nutrition were initiated. The patient’s state continued to deteriorate, and the patient died shortly after.

## Data Availability

The autopsy results are available at the Department of Pathology, County Emergency Hospital, Deva, Romania; contact: patricia.bilei@gmail.com.
